# Association of Paraoxonase 1 Gene Polymorphisms With the Risk of Hepatitis B Virus-related Liver Diseases in a Guangxi Population: A Case-control Study

**DOI:** 10.1097/MD.0000000000002179

**Published:** 2015-12-07

**Authors:** Xianjun Lao, Xiaogang Wang, Yanqiong Liu, Yu Lu, Dongmei Yang, Minyan Liu, Xiaolian Zhang, Chengzhi Rong, Xue Qin, Shan Li

**Affiliations:** From the Department of Clinical Laboratory, First Affiliated Hospital of Guangxi Medical University, Nanning, Guangxi, China.

## Abstract

Paraoxonase 1 (PON1), a liver-induced glycoprotein enzyme responsible for antioxidant defense against reactive oxygen species and anti-inflammatory, has been linked to various cancers. The objective of this study was to explore the association of PON1 rs662 and rs705382 with the risk of chronic hepatitis B (CHB), hepatitis B virus-related liver cirrhosis (LC), and hepatocellular carcinoma (HCC) in patients living in the Guangxi region of southern China.

The PON1 rs662 and rs705382 single-nucleotide polymorphisms (SNPs) were genotyped by polymerase chain reaction–restriction fragment length polymorphism (PCR-RFLP) in 99 CHB patients, 84 LC patients, 258 HCC patients, and 221 healthy controls.

Significant associations with CHB risk were observed for the rs705382 SNP after adjusting for sex, age, ethnicity, smoking, alcohol consumption, and body mass index. When stratified by sex and age, this positive association was significantly strengthened among men and individuals over 40 years old. Moreover, a decreased risk of LC was associated with the rs705382 CG and the combined GG + CG genotypes among women, with borderline statistical significance. In haplotype analyses, the haplotype GA was associated with a 1.68-fold increase in the risk of HCC.

Our results showed that the PON1 rs705382 SNP might be a risk factor for CHB in Guangxi populations.

## INTRODUCTION

Hepotocelluar carcinoma (HCC) is the fifth most commonly diagnosed cancer (782,000 new cancer cases) worldwide and the third most frequent cause of cancer-related death (746,000 cancer deaths) in 2012.^1^ The great majority of HCC cases, regardless of etiology, usually develop in the context of liver cirrhosis (LC).^2^ According to previous studies, an estimated 402,208 new liver cancer cases and 372,079 HCC-related deaths have occurred in China since 2008.^3^ Likewise, HCC cases greatly increased in the United States between 2007 and 2011.^4^ Thus, HCC remains a formidable challenge to medicine despite the great efforts made in its prevention and treatment.

As a multifactorial and complex process, the exact pathogenesis of HCC is still unclear. Hepatitis B virus (HBV) infection is globally the most important etiologic factor of HCC, accounting for an estimated 80% of cases worldwide.^2,5^ Previous cohort studies have indicated that being a chronic carrier of HBV was associated with a 7 to 98-fold higher risk for HCC development.^6^ The risk increases with progressive fibrosis, and is also high in LC patients. In addition to HBV, chronic infection with the hepatitis C virus (HCV), smoking, excessive alcohol consumption, and heavy aflatoxin exposure have also been proposed as risk factors for HCC.^7–9^ However, a large proportion of HCC cases develop among individuals without these known risk factors, suggesting that host genetic factors may play an important role in HCC etiology.^9^

In recent years, the effect of reactive oxygen species (ROS) on hepatic carcinogenesis has been well recognized. It has been suggested that excessive production of ROS may lead to oxidative stress and severe DNA damage, which has been implicated in abnormal cell functioning or even carcinogenesis.^10^ Paraoxonase 1 (PON1) is a liver-induced glycoprotein enzyme that is involved in the mechanism of antioxidant defense against ROS and anti-inflammatory.^11,12^ Mackness et al have reported that PON1 enzymatic activity is correlated with cardiovascular disease, primarily due to the effect of anti-low–density lipoprotein (LDL) oxidative modification.^13^ Additionally, the significant decline of serum PON1 activities has been found to serve as a strong predictor of acute viral hepatitis B, chronic alcoholic hepatitis, liver cirrhosis, and nonalcoholic fatty liver disease (NAFLD).^14–16^ Therefore, single-nucleotide polymorphisms (SNPs) in the PON1 gene may differentially affect the enzymatic activity, possibly contributing to the susceptibility to various diseases.^17^

The human PON1 gene, encoded by the chromosome 7p21–22, contains 9 exons and 8 introns. PON1 presents at least 2 polymorphisms (rs662 and rs854560) located in the coding region and 5 located in the promoter region (rs705379, rs705380, rs705381, rs854571, and rs854572). Of these, rs705379 polymorphism contributed the most (22.8%) and rs854572 polymorphism contributed the least (<1%) of PON1 activity.^18^ The most studied mutation is rs662 (Q192R), which results in a glutamine (Q) to argenine (R) substitution at position 192. The 192Gln variant seems to induce higher PON1 hydrolytic activity towards paraoxon^19,20^ and to be more effective than 192Arg in the prevention of LDL oxidation.^21^ For, several studies have looked at the correlation between the rs662 SNP and the risk of various cancers, such as ovarian epithelial carcinoma,^22^ brain astrocytoma and meningioma,^23^ lymphoma,^24^ and lung cancer,^25^ in various populations. To date, only 1 hospital-based case-control study has investigated the association of PON1 rs662 polymorphism with HCC susceptibility.^26^ Another common polymorphism of PON1 is the rs705382 SNP, which is a C/G substitution at codon 1434 of the 5’-untranslated region (UTR). Previous work revealed that variant allele of rs705382-1434C>G was independently associated with higher levels of PON1 activity and expression.^27^ However, the role of the rs705382 polymorphism in HCC is still unknown.

The aim of this study was to further explore the relationship between 2 previously studied PON1 polymorphisms (rs662 and rs705382) and CHB, HBC-related LC, and HCC susceptibility in Guangxi populations.

## MATERIALS AND METHODS

### Study Population

The present study included 441 HBV-related patients and 221 healthy controls. Patients were periodically recruited at the First Affiliated Hospital of Guangxi Medical University, from April through October of 2014, including 99 CHB patients, 84 HBV-related LC patients, and 258 HBV-related HCC patients. The inclusion and exclusion criteria have been previously described.^28^ Eligible participants were confirmed to have a history of chronic HBV infection (≥6 months). CHB was diagnosed by elevation of the liver function markers alanine aminotransferase (ALT) or aspartate aminotransferase (AST) (>40 IU/mL), as well as by HBV-DNA levels >1 × 10^3^ copies/mL. HBV-LC was diagnosed pathologically or was based on the combination of laboratory tests and radiologic evidence. HBV-HCC was diagnosed based on positive findings obtained from ultrasonography, computed tomography (CT), and magnetic resonance imaging (MRI), and/or by pathological examination. A significant increase in serum α-fetoprotein (AFP) (>400 ng/mL) was also determined to indicate HBV-HCC. Patients were excluded from the current study if they had coinfection with other hepatitis virus (hepatitis A/C/D/E virus); alcoholic liver disease, autoimmune hepatitis, or primary biliary cirrhosis; any concomitant malignant cancers; or a family history of HCC, or a history of autoimmune diseases such as systemic lupus erythematosus (SLE) and rheumatoid arthritis (RA). Controls were randomly selected from healthy individuals who attended the same hospital for a routine physical examination during the patient recruitment periods. The selection criteria for controls were absence of any malignancy or other hepatic illness, including HBV infection. All participants were from the Guangxi district. At recruitment, written informed consent was obtained from each participant. Additionally, blood samples and demographic characteristics, including age, sex, ethnicity, tobacco and alcohol use, and body mass index (BMI), were also collected by the interviewers. This study was approved by the ethics committee of the First Affiliated Hospital of Guangxi Medical University.

### DNA Extraction and PON1 Polymorphism Genotyping

**FIGURE 1:**
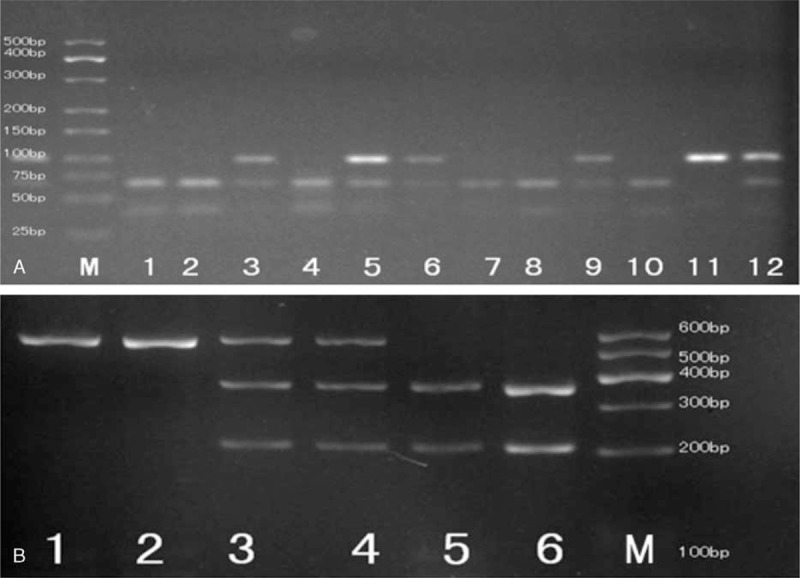
PCR-RFLP assay for analyzing the rs662 and rs705382 polymorphisms of the PON1 gene. (A) rs662-lanes M: DNA marker; lanes 1, 2, 4, 7, 8, and 10 show GG genotype; lanes 3, 5, 6, 9, and 12 show AG genotype; lane 11 shows AA genotype. (B) rs705382-lanes M: DNA marker; lanes 1 and 2 show GG genotype; lanes 3 and 4 show CG genotype; lanes 5 and 6 show CC genotype. PCR-RFLP = polymerase chain reaction–restriction fragment length polymorphism, PON1 = paraoxonase 1.

Genomic DNA was extracted from 2-mL peripheral venous blood by a high-salting-out method using phenol-chloroform. The PON1 rs662 and rs705382 SNPs were genotyped by polymerase chain reaction–restriction fragment length polymorphism (PCR-RFLP), without knowledge of the case or control status. The PCR was carried out in 1.0 μL of each primer, 12.5 μL of Green PCR Master Mix (Shanghai Sangon Biotech Co., Ltd., Shanghai, China), 9.5 μL of sterilized deionized water, and 2.0 μL of template DNA per reaction. The forward and reverse primers used were rs662-F 5’-TATTGTTGCTGTGGGACCTGAG-3’ and R 5’-CACGCTAAACCCAAATACATCTC-3’,^29^ rs705382-F 5’-GAGAGGGAAAGTGGTCAGCT-3’ and R 5’-GAAGTGTGAGTTTGGGCAGG-3’. The PCR reaction for rs662 included a 5-min preincubation step at 95°C, followed by 40 cycles of 45 seconds at 95°C, 45 seconds at 59°C, 45 seconds at 72°C, and then a final 10-min extension step at 72°C. For rs705382, it included a 5-min preincubation step at 95°C, followed by 25 cycles of 39 seconds at 95°C, 30 seconds at 56°C, 30 seconds at 72°C, and then a final 10-min extension step at 72°C. After amplification, all products were separated on 2.5% agarose gel and subsequently stained with ethidium bromide to visualize the bands (Fig. 1). To control the quality of the PCR reaction, a negative control was also performed in each genotyping assay. In addition, a 10% random sample was send to Sangon Biotech Company for genotyping in duplicates, and the reproducibility was 100%.

### Statistical Analysis

The Hardy–Weinberg equilibrium (HWE) was assessed by a goodness-of-fit chi-square test for genotypes in the control groups. For baseline data, 1-way analysis of variance (ANOVA) tests were used for the categorical variables, and Student *t* tests were used for the continuous variables to compare the differences in demographic characteristics between patients and controls. Genotype and allele frequencies among different groups were compared using the chi-square test and Fisher exact test, when appropriate. Logistic regression analysis, adjusted for age, sex, tobacco smoking, alcohol consumption, and BMI, was utilized to calculate the adjusted odds ratios (ORs) and 95% confidence intervals (CIs). Further stratified analyses were conducted according to age and sex. The haplotype construction was performed using SHEsis software (http://analysis.bio-x.cn/myAnalysis.php).^30^ A probability level of less than 0.05 was judged as statistically significant. All statistical analyses were performed using SPSS, version 13.0 (SPSS Inc., Chicago, IL).

## RESULTS

### Characteristics of the Study Population

The basic demographics of patients and controls are summarized in Table 1. Briefly, the CHB patients were, on average, significantly younger than the healthy control, LC, and HCC patients (*P* < 0.001). The HCC patients were predominantly men, tobacco smokers, and alcohol drinkers (*P* < 0.001). However, the healthy control group consisted largely of nonsmokers and nondrinkers, which indicated that the controls seemed to have a healthier lifestyle in comparison with the HCC patients. Apart from this, most of the healthy controls were from the Zhuang ethnic group, whereas the HBV patients were primarily from the Han ethnic group (*P* = 0.044). The mean BMI in the control individuals was similar to that in the HBV patients (*P* = 0.210). In healthy controls, the observed genotype frequencies of rs662 and rs705382 were both in agreement with HWE (*P* = 0.437 for rs662 and *P* = 1.000 for rs705382) (Table 2).

**TABLE 1:**
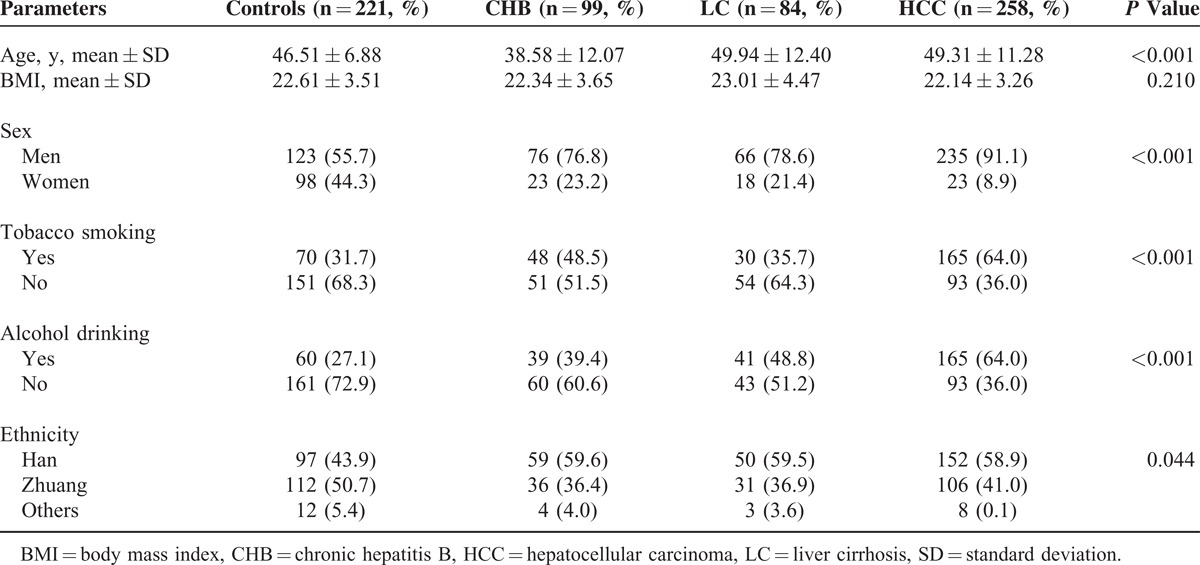
Clinical and Epidemiological Data for the Patient and Control Groups

**TABLE 2:**
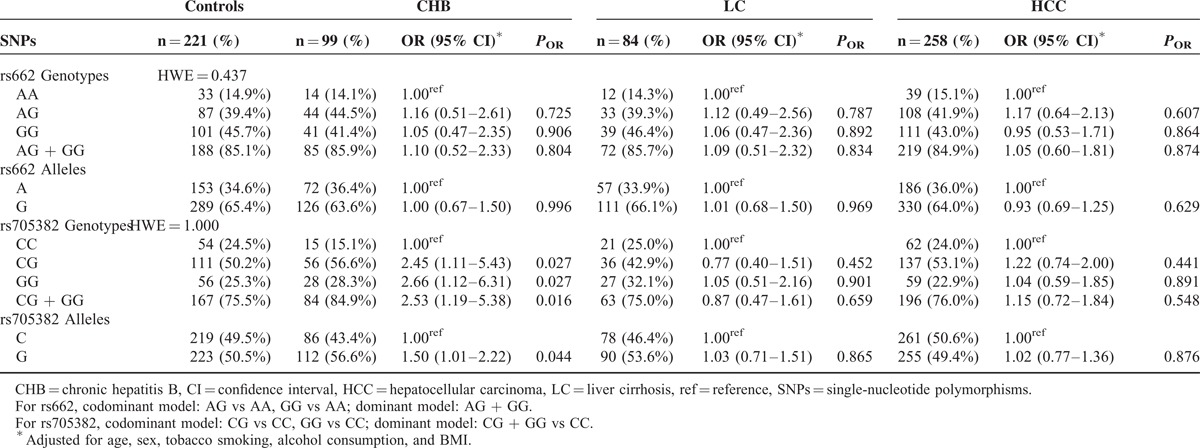
Genotype and Allele Frequencies of Candidate SNPs in Patients and Control Individuals

### Association Analysis of Candidate SNPs with CHB, HBV-related LC, and HCC Risk

Table 2 shows the genotype and allele frequencies of the 2 candidate SNPs within the PON1 gene for the patient and control groups. Distributions of the rs662 and rs705382 genotypes and alleles were then compared across the groups, and no statistically significant differences were observed (both *P* > 0.05).

Associations between the PON1 rs662 polymorphism and CHB, HBV-related LC, and HCC risk were assessed using logistic regression analyses adjusted for sex, age, ethnicity, smoking, alcohol consumption, and BMI, and no significant associations were observed in any genetic models (Table 2). However, logistic regression analyses for the PON1 rs705382 polymorphism indicated that individuals carrying the rs705382 G allele were at moderately increased risk of CHB when compared with those carrying the C allele (adjusted OR 1.50, 95% CI 1.01–2.22, *P* = 0.044; Table 2). Similarly, we also found that both the rs705382 GG and CG carriers had more than a 2-fold risk of CHB (GG vs CC genotype: adjusted OR 2.66, 95% CI 1.12–6.31, *P* = 0.027; CG vs CC genotype: adjusted OR 2.45, 95% CI 1.11–5.43, *P* = 0.027) relative to the CC carriers. In the combined analyses, the GG + CG genotype was associated with a significantly increased risk of CHB, with adjusted OR of 2.53 (95% CI 1.19–5.38, *P* = 0.016).

We further examined the associations between the 2 PON1 SNPs and CHB, HBV-related LC, and HCC risk by stratifying each study participant into subgroups according to age and sex. The results suggested that men with rs705382 GG genotype and G allele exhibited an increased risk of CHB (GG vs CC genotype: adjusted OR 3.59, 95% CI 1.23–6.31, *P* = 0.020; G vs C allele: adjusted OR 1.76, 95% CI 1.07–2.88, *P* = 0.025; Table 3). Interestingly, compared with the rs705382 CC genotype, a marginally decreased risk of LC was associated with the CG and the combined GG + CG genotypes among women (CG vs CC genotype: adjusted OR 0.26, 95% CI 0.07–0.98, *P* = 0.047; dominant model: adjusted OR 0.28, 95% CI 0.08–0.96, *P* = 0.042). On the contrary, neither the allele frequencies nor the genotype distributions of the rs662 SNP showed impacts on men or women in both the case and control groups (Table 3). In addition, among individuals aged 40 years or older, strong evidences of associations between CHB and the rs705382 variant genotypes occur, with adjusted OR of 10.336 for GG versus CC (95% CI 1.27–84.11, *P* = 0.029), adjusted OR of 11.54 for CG versus CC (95% CI 1.47–90.68, *P* = 0.020), and adjusted OR of 11.05 for the dominant model (95% CI 1.44–84.76, *P* = 0.021) (Table 4). Similar to the stratified results for sex, negative results were found for rs662 polymorphism in both older individuals (≥40 years old) and younger individuals (<40 years old).

**TABLE 3:**
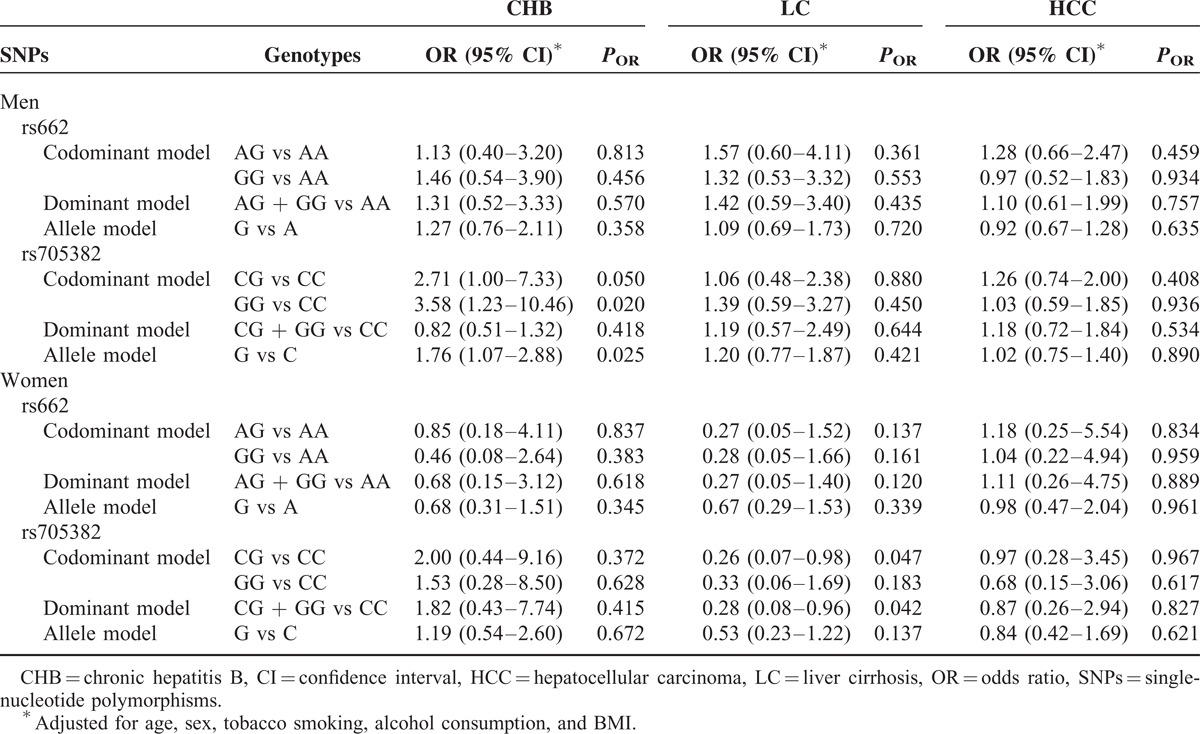
Stratified Effects of PON1 rs662 and rs705382 Polymorphisms on CHB, LC, and HCC Risk Estimated by Sex

**TABLE 4:**
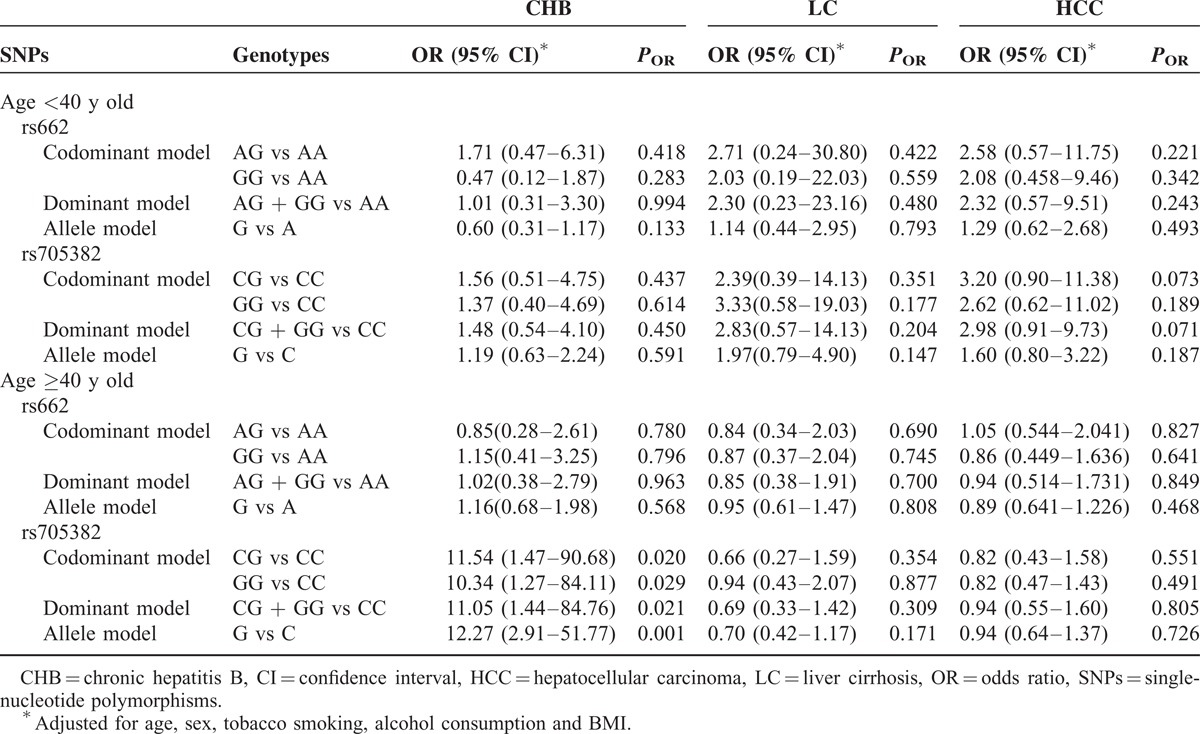
Stratified Effects of PON1 rs662 and rs705382 Polymorphisms on CHB, LC, and HCC Risk Estimated by Age

The genetic background of PON1 polymorphisms may vary between different races and populations. Therefore, the allele and genotype frequencies of the 2 SNPs in the control groups were compared with those previously reported in the Haplotype Map (HapMap) project (http://www.ncbi.nlm.nih.gov/snp/) and several human genetic association studies.^31–33^ As is shown in Table 5, the distribution of the 2 SNPs in the present study is significantly different from that in CEU (Utah residents with northern and western European ancestry) and YRI (Yoruba in Ibadan). For the rs662 SNP, the frequencies of the GG genotype and G allele in CEU population are significantly lower, whereas it is higher in the YRI population when compared with our data. In contrast, the GG genotype and G-allele distributions of the rs705382 SNP are significantly higher and lower in the CEU and YRI populations, respectively.

**TABLE 5:**
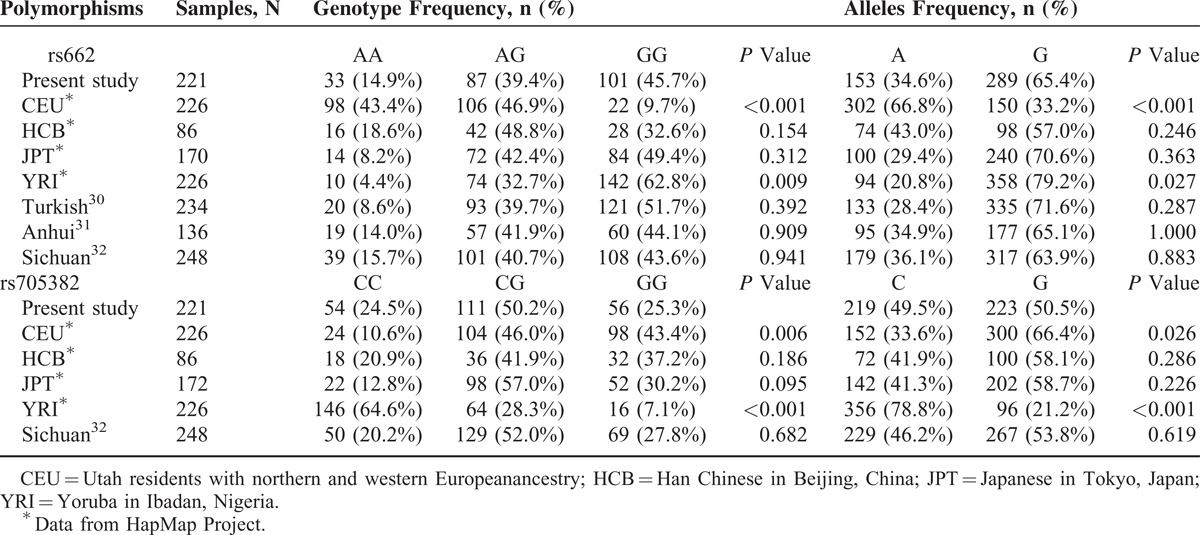
Comparison of Genotype and Allele Frequencies in the Healthy Control Participants of Our Study and That From the HapMap Project

### Haplotype Analysis of Associations Between PON1 Polymorphisms and CHB, HBV-related LC, and HBV-related HCC

We performed further the haplotype analysis to evaluate the linkage strength of nearby SNPs located in the same chromosome regions. A total of 4 two-locus haplotypes (CA, CG, GA, and GG) were analyzed. We identified that GG was the most common haplotype in both patients and controls. CA was the next most common in healthy controls, CHB, and LC patients, whereas CG was in second place for HCC patients (Table 6). The frequency of the haplotype GA was significantly associated with increased HCC risk (OR 1.68, 95% CI 1.08–2.60, *P* = 0.020).

**TABLE 6:**
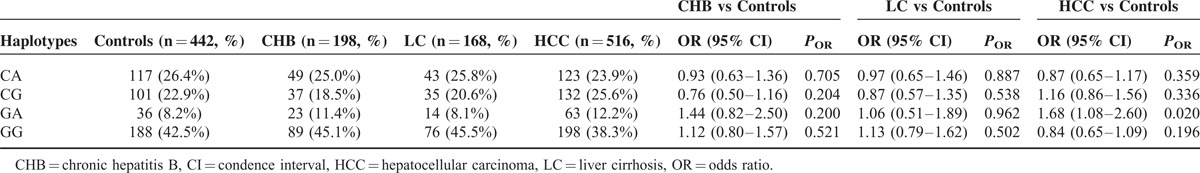
Haplotype Analysis of PON1 rs662 and rs705382 Polymorphisms With the Risk of CHB, LC, and HCC

## DISCUSSION

In this study, we investigated the association of PON1 gene SNPs (rs662 and rs705382) with risk of CHB, HBV-related LC, and HCC in the Guangxi population. Our results demonstrate that, for the overall population, the candidate rs705382 SNP was statistically significantly related with greater risk of CHB, but not in LC or HCC. When stratified by sex and age, significant associations with increased risk of CHB were observed for the rs705382 SNP in men and older individuals (≥40 years old), whereas a marginally decreased risk of HBV-related LC was found for the variant genotype of rs705382 among women. Haplotype analysis of the 2 candidate SNPs revealed that the haplotype GA served as an increase risk factor for HCC.

The pathogenic mechanism linking oxidative stress and hepatocarcinogenesis has previously been studied.^34,35^ PON1, one member of the PON family, plays a crucial role in antioxidant defense and anti-inflammatory.^11,12^ Sun et al^36^ reported that the expression levels of PON1 protein in patients with LC and HCC were significantly lower than that in the healthy controls. Therefore, it is easily inferred that the significant decline of serum PON1 concentrations or activity can profoundly affect liver disease status. According to previous results, genetic mutations like promoter polymorphisms are the most important factors regarding the variations in PON1 enzyme activity between individuals. Several studies have reported a functional significance of PON1 gene polymorphisms. It has been suggested that the variant alleles of PON1 rs662 and rs705382 seem to induce higher PON1 activity.^19,20,27^ However, there are fewer studies investigating the association of the PON1 rs662 and rs705382 polymorphism with CHB and LC, as well as with HCC.

In 2013, based on 217 confirmed cases and 217 cancer-free controls, Akkiz et al^26^ firstly reported the association of PON1 rs662 polymorphisms with HCC risk in the Turkish population. In that study, the variant allele frequencies were 0.28 and 0.27 for HCC cases and controls, respectively. Further statistical analysis showed that the rs662 polymorphisms have no direct effect on the risk for developing HCC, which is in agreement with our results. Furthermore, there were 2 studies investigating the associations of the PON1 rs705382 polymorphism with ischemic stroke (IS) and sporadic amyotrophic lateral sclerosis (SALS), respectively. The study on the association between rs705382 SNP and ischemic stroke was conducted by Kim et al^27^ in 2009, and involved 86 Korean patients with IS. According to the results, the rs705382 G allele was observed with a little lower frequency in cases than in controls. A general linear model revealed that the rs705382-1434G was related with higher PON1 activity and acted as a risk factor for IS in the Korean population.^27^ In contrast, Chen et al^31^ assessed 373 SALS patients and 248 controls from Southwest China, and the rs705382 polymorphism did not seem to contribute to the risk for developing SALS in that study population, as evidenced by the absence of statistical differences in allele frequencies between the control and SALS group, and by the association analysis of rs705382 SNP with SALS risk. Nevertheless, either PON1 rs662 or rs705382 SNP was investigated in these studies. Few studies so far have estimated the influences of both the PON1 rs662 and rs705382 polymorphisms on CHB, LC, and HCC risks. The current study was conducted to investigate the association between 2 SNPs (rs662, rs705382) of the PON1 gene, and CHB, LC, and HCC risks in a substantial number of cases and controls (99 CHB patients, 84 LC, 258 HCC, and 221 healthy controls), which greatly increased statistical power of the study when compared with previous studies. In our study, the rs705382-1434G allele was distributed as follows: 0.57 in CHB, 0.54 in LC, 0.51 in HCC, and 0.51 in healthy controls. Although no significant differences were found in the allele frequencies between patients and controls, the rs705382 SNP was associated with increased risk of CHB, after adjusting for all conventional risk factors. It is possible that these inconsistent findings may be explained by the differences in studied population, sample size, and other potential risk factors that influence the effects of genetic variants on disease risk.

On the basis of the subgroup analysis by sex and age, our observations suggest that there are age and sex-related differences in the correlation between the PON1 rs705382 polymorphism and CHB risk. Individuals with the rs705382 GG genotype had a significantly high risk of CHB among men and people over 40 years old, but no such results were observed among women and people younger than 40 years old. Notably, compared with those of the CC genotype, women carrying the rs705382 CG and the combined CG + GG genotypes were at increased risk of LC. However, this association only bordered on statistical significance and the result should be interpreted cautiously. Previous studies have shown a considerable decrease of PON1 activity with advancing age,^37,38^ and this leads to increased risk of disease among older individuals. On the contrary, Hernandez et al^39^ reported that women had significant lower levels of PON1 among the Spanish population, suggesting that women may be more susceptible to diseases. Nevertheless, this is not in line with our results. Considering the distinct genetic background and differences in exposure to environmental risk factors between the Chinese and Spanish populations, further similar studies with a larger number of patients should be performed for confirmation.

Analysis of multiple linked SNPs tend to be more powerful for providing robust results than individual SNPs.^40^ Meanwhile, information is lacking regarding PON1 haplotypes and HCC risk. Consequently, haplotype analysis is to be preferred while evaluating the relationship of PON1 polymorphisms with HCC risk by using the SHEsis software. We observed that the haplotype GG (carrying all variants) was the most prevalent one in both the patient and control groups; however, the haplotype GA (carrying 1 variant and 1 wild-type allele) was associated with a 1.68-fold increase in the risk of HCC.

To the best of our knowledge, the present study is the first to assess the potential implications of the PON1 gene rs662 and rs705382 for susceptibility to CHB, HBV-related LC, and HCC. Despite the strengths and biological plausibility of the associations observed in this study, there are still several limitations need to be addressed. Firstly, the study participants were all recruited from the Guangxi district, and they may not be representative of the entire Chinese population. Data should be applied cautiously to other ethnic groups. Additionally, because of the lack of dietary exposure assessment of aflatoxin, we have not carried out the gene–environment interaction analyses of aflatoxin exposure and PON1 candidate SNPs in risk estimates of HCC. Follow-up studies are needed to examine the detailed molecular interactions between PON1 polymorphisms and aflatoxin exposure.

In conclusion, our results showed that the PON1 rs705382 SNP was associated with the increased risk of CHB in a Guangxi population, especially in men and individuals over 40 years old. Larger prospective studies with detailed information about aflatoxin exposure are warranted to validate our findings.
